# No metabolic effects of mustard allyl-isothiocyanate compared with placebo in men

**DOI:** 10.3945/ajcn.116.148395

**Published:** 2017-10-25

**Authors:** Mirjam Langeveld, Chong Yew Tan, Maarten R Soeters, Samuel Virtue, Laura PE Watson, Peter R Murgatroyd, Graeme K Ambler, Santiago Vidal-Puig, Krishna V Chatterjee, Antonio Vidal-Puig

**Affiliations:** 1 University of Cambridge Metabolic Research Laboratories, Wellcome Trust-Medical Research Council, Institute of Metabolic Science, and; 2 National Institute for Health Research/Wellcome Trust Clinical Research Facility, Addenbrookes Hospital, Cambridge, United Kingdom;; 3 Department of Applied Statistics and Operational Research and Quality, Technical University of Valencia, Valencia, Spain; and; 4 South East Wales Vascular Network, Aneurin Bevan University Health Board, Royal Gwent Hospital, Newport, United Kingdom

**Keywords:** mustard, allyl-isothiocyanate, energy expenditure, thermogenesis, thermogenic food

## Abstract

**Background:** Induction of nonshivering thermogenesis can be used to influence energy balance to prevent or even treat obesity. The pungent component of mustard, allyl-isothiocyanate (AITC), activates the extreme cold receptor transient receptor potential channel, subfamily A, member 1 and may thus induce energy expenditure and metabolic changes.

**Objective:** The objective of our study was to evaluate the potential of mustard AITC to induce thermogenesis (primary outcome) and alter body temperature, cold and hunger sensations, plasma metabolic parameters, and energy intake (secondary outcomes).

**Design:** Energy expenditure in mice was measured after subcutaneous injection with vehicle, 1 mg norepinephrine/kg, or 5 mg AITC/kg. In our human crossover study, 11 healthy subjects were studied under temperature-controlled conditions after an overnight fast. After ingestion of 10 g of capsulated mustard or uncapsulated mustard or a capsulated placebo mixture, measurements of energy expenditure, substrate oxidation, core temperature, cold and hunger scores, and plasma parameters were repeated every 30 min during a 150-min period. Subjects were randomly selected for the placebo and capsulated mustard intervention; 9 of 11 subjects received the uncapsulated mustard as the final intervention because this could not be blinded. After the experiments, energy intake was measured with the universal eating monitor in a test meal.

**Results:** In mice, AITC administration induced a 32% increase in energy expenditure compared with vehicle (17.5 ± 4.9 J · min^−1^ · mouse^−1^ compared with 12.5 ± 1.2 J · min^−1^ · mouse^−1^, *P* = 0.03). Of the 11 randomly selected participants, 1 was excluded because of intercurrent illness after the first visit and 1 withdrew after the second visit. Energy expenditure did not increase after ingestion of capsulated or uncapsulated mustard compared with placebo. No differences in substrate oxidation, core temperature, cold and hunger scores, or plasma parameters were found, nor was the energy intake at the end of the experiment different between the 3 conditions.

**Conclusion:** The highest tolerable dose of mustard we were able to use did not elicit a relevant thermogenic response in humans. This trial was registered at www.controlled-trials.com as ISRCTN19147515.

## INTRODUCTION

Mild cold exposure induces nonshivering thermogenesis (NST), which is at least partially mediated by the activation of brown adipose tissue (BAT). This specialized thermogenic organ produces heat from glucose and lipid oxidation by uncoupling the electron transport chain from ATP production (1–3). When maintained over a long period of time, this small increase in energy expenditure can alter the energy balance to prevent the development of obesity or even mediate modest weight loss. Even mild cold exposure is perceived as unpleasant (4), however, and the achievement of continuous mild cold exposure has practical issues. As such, alternative methods to induce NST are desirable.

The concept of the use of thermogenic food components to increase energy has long existed and has received renewed attention with the discovery of BAT-mediated NST in humans (5). Examples of thermogenic food components include caffeine, catechin, capsaicin, capsiate, and cinnamaldehyde (6–9).

The cold-sensing receptors, which are present on the nerve endings of skin and in mucous membranes, have been characterized during the past 2 decades. They belong to the family of transient receptor potential channels (TRPs). Different TRPs are sensitive to different temperature ranges: 18–24°C is sensed by TRP subfamily M, member 8 (TRPM8) and temperatures <18°C are sensed by TRP subfamily A, member 1 (TRPA1) (10, 11). Cold sensing via these receptors initiates a cascade of responses resulting in changes in behavior, insulation, and metabolic rate. Allyl-isothiocyanate (AITC), the pungent component of mustard, activates TRPA1. In mice, AITC activates BAT and at the same time causes vasoconstriction in the tail, which is consistent with a response to cold exposure (12). AITC mixed in a high-fat diet also reduces weight gain and improves insulin sensitivity (13). In addition, the inhibition of lipogenesis by AITC metabolites may have antiobesity effects (14).

Little is known about the metabolic response to cold receptor–activating food components in humans. A study of the effects of different food components on postprandial metabolic rate showed that mustard tends to increase metabolic rate (15); however, the thermogenic food components were mixed in a meal, which in itself has a thermogenic effect (16).

The objective of the present study was to evaluate the potential of mustard AITC to induce thermogenesis (primary outcome) and alter body temperature, cold and hunger sensations, plasma metabolic parameters, and energy intake (secondary outcomes). We conducted a proof-of-principle study in mice, measuring energy expenditure in response to AITC treatment. Next, we studied the effect of mustard on energy expenditure, substrate oxidation patterns, temperature, and sensations of cold and hunger in humans. We hypothesized that AITC would increase energy expenditure. A study arm in which the mustard was capsulated was included to determine whether any metabolic response was elicited by the stimulation of cold receptors in the mouth or further down the gastrointestinal tract, and to correct for the potential stress effect of the strong taste.

## METHODS

### Ethics

All of the animal protocols used in this study were approved by the Home Office (United Kingdom) and the University of Cambridge. All of the human subjects provided written informed consent, and the study conformed to the standards set by the latest revision of the Declaration of Helsinki. The study received approval from the Cambridge Central East of England Research Ethics Committee. The trial was registered at www.controlled-trials.com as ISRCTN19147515.

### Measurement of thermogenic response in mice

The thermogenic response to subcutaneous injection with vehicle, norepinephrine, or AITC was assessed by indirect calorimetry under anesthesia. Indirect calorimetry was performed in an Oxymax calorimetry chamber (Columbus Instruments), which had a 2.7-L capacity and was housed within a large temperature-controlled cabinet. Room air to the chamber was passed through a heat exchanger to warm it to 30°C. The temperature within the calorimetric chambers was continuously monitored and fixed at 30°C. Oxygen consumption (VO_2_), carbon dioxide production (VCO_2_), and respiratory exchange ratio (RER) (VCO_2_/VO_2_) were measured with the use of a custom-built oxygen and carbon dioxide monitoring system. Energy expenditure was calculated from VO_2_ and VCO_2_ with a modified Weir equation. Airflow rates were set at 400 mL/min. Measurements of oxygen and carbon dioxide concentrations in the room air and the air leaving each cage were taken every 4 min. Mice were placed in the calorimetric chamber following intraperitoneal injection of sodium pentobarbital (90 mg/kg). Baseline gas exchange was recorded once steady state was achieved (≥3 consecutive stable measurements). Mice were then injected with either 1 mg norepinephrine/kg (Sigma), 5 mg AITC/kg, or vehicle phosphate-buffered saline, and returned to the calorimetry chamber. Maximal VO_2_rates were achieved typically within 12–16 min postinjection and were defined as 3 stable consecutive readings.

### Measurement of thermogenic response in humans

#### Subjects

Healthy volunteers were recruited through local advertisement in the East Anglia region of the United Kingdom. We recruited 6 men and 5 women who were nonsmokers, aged between 18 and 65 y, without any known medical conditions, and not taking any medications or supplements likely to influence energy expenditure. All of the subjects provided written informed consent. Parts of this study (e.g., data of the 24°C control visit) have been published elsewhere (4). Before the start of the study, we investigated what the maximal dose of mustard to be taken orally without inducing nausea would be and found that it was 10 g.

#### Study outline

**FIGURE 1:**
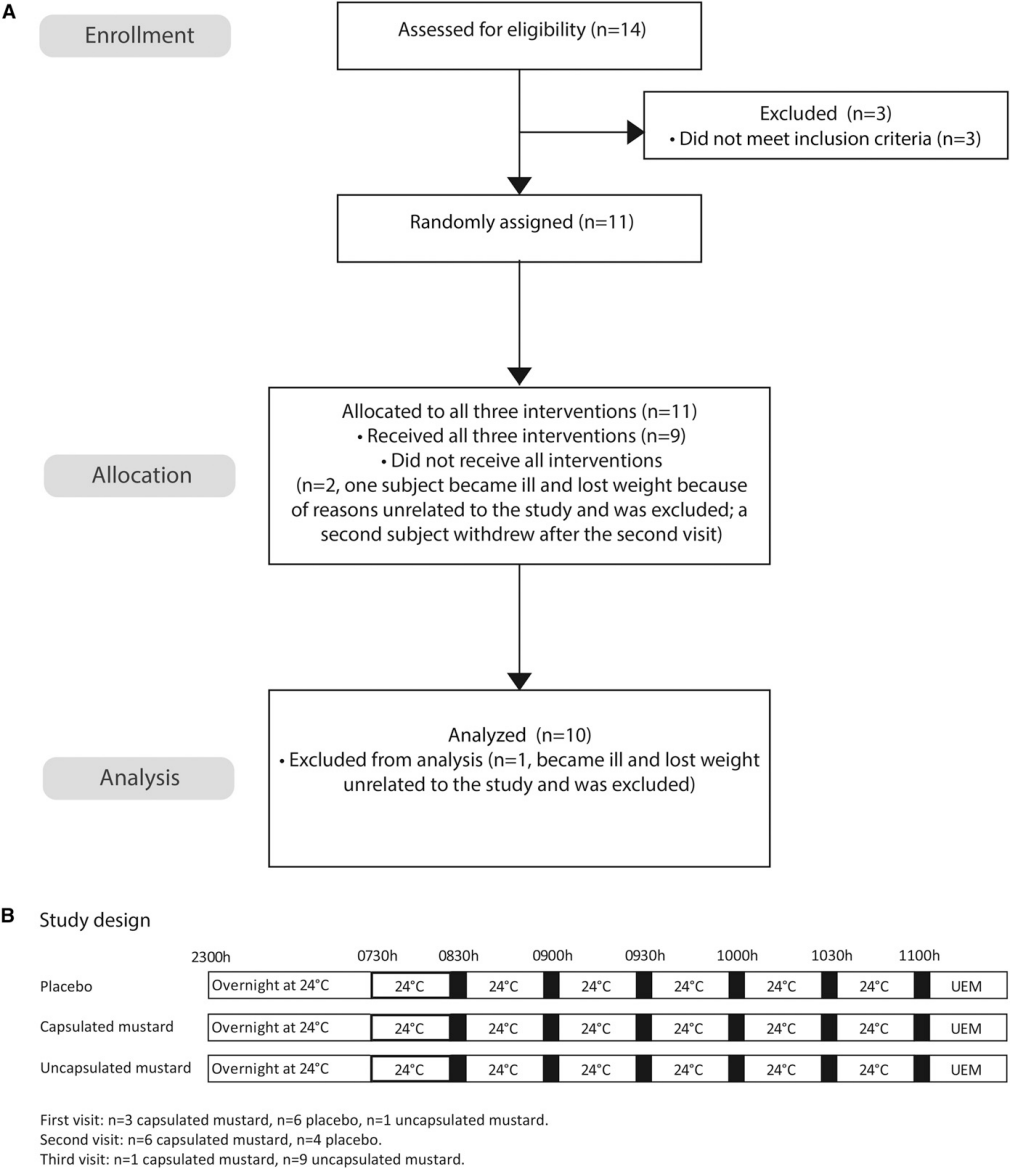
Study outline. Consolidated Standards of Reporting Trials flowchart (A). (B) Study design. Black bars represent measurements including indirect calorimetry, cold and hunger scores, and blood tests. UEM, universal eating monitor.

The outline of the study design is depicted in **Figure 1**. Subjects were studied on 3 separate days under different conditions: ingestion of uncapsulated mustard, ingestion of capsulated mustard, or ingestion of capsulated placebo mixture. All of the experiments were performed at thermoneutrality (24°C). 

The study was a crossover design. Subjects received the placebo and packaged mustard as an initial intervention (coin flip performed by the investigators), and subjects but not the investigators were blinded for the intervention. Uncapsulated mustard was administered as the final intervention in 9 of 10 participants and in 1 subject as the initial intervention. Participants could not be blinded for this intervention. Subjects were asked to refrain from strenuous physical activity, alcohol, and caffeine for 24 h before their visit. Each participant arrived at the National Institute for Health Research/Wellcome Trust Clinical Research Facility at ∼1600 on day 0 and remained until 1400 on day 1. Height, weight, and body composition [dual-energy X-ray absorptiometry (software version 12.2; GE Lunar Prodigy GE Health Care)] were measured. 

At 1800, a standardized dinner was served. The energy content of the meal was one-third of a participant’s daily requirements estimated from predicted resting metabolic rate through the use of the Schofield equation, multiplied by an activity factor of 1.35. Meal composition was 30–35% fat, 12–15% protein, and 50–55% carbohydrate by energy. The participants retired to bed in the temperature-controlled room (24°C) at 2300 and were provided with standardized lightweight clothing and bedding. Participants were awakened the next morning at 0700 and, after use of the toilet, returned to bed and remained in a semisupine position (upper part of the bed at a 45° angle) without bedding. Next, participants ingested a temperature-monitoring pill (VitalSense; Respironics). Baseline indirect calorimetry, cold and hunger scores, and blood tests were taken at 0730, and then either capsulated mustard (10 g), capsulated placebo mixture (10 g), or a spoonful of mustard (10 g) was ingested by subjects. The mustard contained ∼1.6 mg AITC/g mustard (Colman’s Mustard). The placebo mixture was made by combining tomato ketchup, olive oil, and water to match the energy content, macronutrient composition, and volume of the mustard.

During the experiment, indirect calorimetry, cold and hunger scores, and blood tests were repeated every 30 min (Figure 1). Between measurements, the subjects were allowed to read or watch television but were confined to bed. Blood samples were taken via an indwelling venous catheter. Afterward, a universal eating monitor (UEM) was used to assess parameters related to appetite and food intake.

#### Indirect calorimetry

Resting energy expenditure was measured by ventilated canopy respiratory gas exchange monitor (GEMNutrition), with participants in the supine position. Measurements were recorded at 30-s intervals for 20 min. All of the participants were asked to remain awake and motionless for the duration of the measurement. Energy expenditure was calculated from the macronutrient respiratory quotients and energy equivalents of oxygen published by Elia and Livesey (17).

#### Core temperature

We measured core temperature throughout the experiment using a temperature-monitoring pill.

#### Cold and hunger scores

Participants were asked to rate on a 1–10 scale the sensation of cold in the whole body and in the hands separately, with 1 being not at all cold and 10 being the coldest one had ever felt. They also were asked to rate on a 1–10 scale the degree of hunger, with 1 being not hungry at all and 10 being the most hungry one had ever felt.

#### Blood biochemistry

Glucose was measured through the use of the hexokinase method on a Siemens Dimension RXL AutoAnalyzer; reagents and calibrators were purchased from Siemens. Nonesterified fatty acids were measured with the Roche Free Fatty Acids kit. Free thyroxin was measured by time-resolved fluorescence immunoassay on an AutoDELFIA analyzer (PerkinElmer). Cortisol was measured by fluorescence immunoassay on the Siemens Centaur Autoanalyzer. At minimum, 2 quality control samples were run in each assay.

#### UEM

A UEM (Sussex Meal Patterning System) was used to measure energy intake in a homogenous test meal (e.g., pasta) containing normal energy percentage ratios (∼30% carbohydrates, ∼30% protein, and ∼40% fat). Test meal intake was continuously monitored with the UEM equipment (18). Food was served and eaten from a plate placed on a weighing scale connected to a computer to generate data on the amount eaten and time spent eating.

### Statistical analysis

#### Mouse experiment

The maximum thermogenic capacity was defined as the mean of the highest 3 measurements after injections with norepinephrine, AITC, or vehicle. Comparison between the different conditions was made with the Student’s *t* test in Microsoft Excel. Data are presented as means ± SDs.

#### Human experiments: power calculation

We attempted to detect the effect of mustard ingestion on energy expenditure that had at least the magnitude of the effect of mild cold exposure. In our previously published study, energy expenditure for 150 min, after subtracting baseline energy expenditure at thermoneutrality, was 47.9 ± 43.8 kJ at mild cold exposure (18°C) and 6.2 ± 22.7 kJ at thermoneutrality (24°C; *P* = 0.01) (4). The minimum sample size needed to detect this difference in the present study was 7 subjects for each condition (thermoneutrality, capsulated mustard ingestion, and uncapsulated mustard ingestion), a desired α of 0.05, and a desired power of 0.8.

#### Results analysis

Analysis was performed by use of SPSS version 23 (IBM). An intention-to-treat analysis was conducted. Missing data implementation was accomplished via trimmed scores regression (19). Time series data were analyzed with double repeated-measures ANOVA. Each ANOVA model was built with “time” and “intervention” as within-subject effects. A significant “intervention” effect and its interaction with time is interpreted as a significant effect of mustard ingestion on the rate of change over time.

Each term in the ANOVA model was analyzed for sphericity (Mauchly’s test), and if found to be violated, then within-subject effects were determined by the Greenhouse-Geisser test. For all of the statistical tests, *P* values <0.05 were considered significant. In the case of a significant effect on the qualitative factor (intervention), CIs on the means with Bonferroni adjustment are considered; however, it must be noted that in our analysis, the effect of the intervention was not significant. In the case of a significant quantitative factor (time), we analyzed the nature of the response function (linear, quadratic) through the use of orthogonal contrasts. All paired data were analyzed by the Student’s *t* test. Correlations were assessed with a Pearson’s test. Original data, without imputation, are presented as means ± SDs.

## RESULTS

### Mice

**FIGURE 2:**
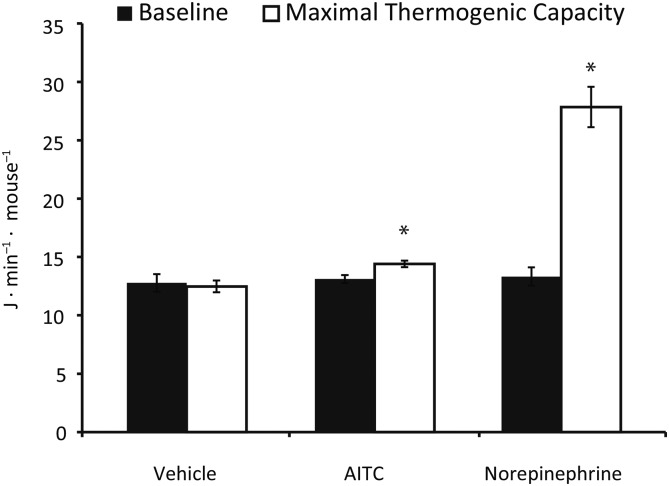
Effect of high-dose AITC on energy expenditure in mice. Mice were 12-wk-old male C57BL/6J, saline-injected controls (vehicle, *n* = 6), norepinephrine treated (*n* = 5), and AITC treated (*n* = 6). Bars represent means and error bars represent SEMs, *P < 0.05 (maximum thermogenic capacity after injection with norepinephrine compared with vehicle and AITC compared with vehicle), with the use of the Student’s *t* test. AITC, allyl-isothiocyanate.

Baseline metabolic rates were not different between the intervention groups (norepinephrine baseline: 13.3 ± 1.8 J · min^−1^ · mouse^−1^, AITC baseline: 13.1 ± 0.9 J · min^−1^ · mouse^−1^, and vehicle baseline: 12.8 ± 1.9 J · min^−1^ · mouse^−1^; *t* test norepinephrine compared with vehicle; *P* = 0.64, AITC compared with vehicle: *P* = 0.74) (**Figure 2**). Maximal thermogenic capacity after injecting norepinephrine and after injecting AITC was significantly higher than after injecting vehicle (norepinephrine maximum: 27.9 ± 3.9 J · min^−1^ · mouse^−1^, AITC maximum: 17.5 ± 4.9 J · min^−1^ · mouse^−1^, and vehicle maximum: 12.5 ± 1.2 J · min^−1^ · mouse^−1^, norepinephrine compared with vehicle: *P* < 0.01, AITC compared with vehicle: *P* = 0.03) (Figure 2).

### Humans

Subject characteristics are summarized in **Table 1** and in the Consolidated Standards of Reporting Trials flowchart and study design in Figure 1. Of the 11 participants randomly selected, 1 became ill and lost weight shortly after the first study visit and was then excluded from the study. One of the remaining 10 subjects withdrew after the second visit. The order of the interventions in the 10 remaining subjects is outlined in Figure 1.

**TABLE 1:** Subject characteristics^1^

	Men (*n* = 6)	Women (*n* = 5)
Age, y	41.6 ± 12.2	33.7 ± 6.9
BMR, J/min	3845 ± 463	3808 ± 197
Height, m	1.74 ± 0.1	1.65 ± 0.1
Weight, kg	68.5 ± 6.4	62.6 ± 4.1
BMI, kg/m^2^	22.5 ± 1.5	22.9 ± 0.9
Fat, kg	15.5 ± 5.0	20.2 ± 2.4
Lean, kg	50.5 ± 4.5	39 ± 2.2
FFM, kg	53.0 ± 5.2	41.5 ± 2.4

1 Values are means ± SDs. BMR, basal metabolic rate; FFM, fat-free mass.

**FIGURE 3:**
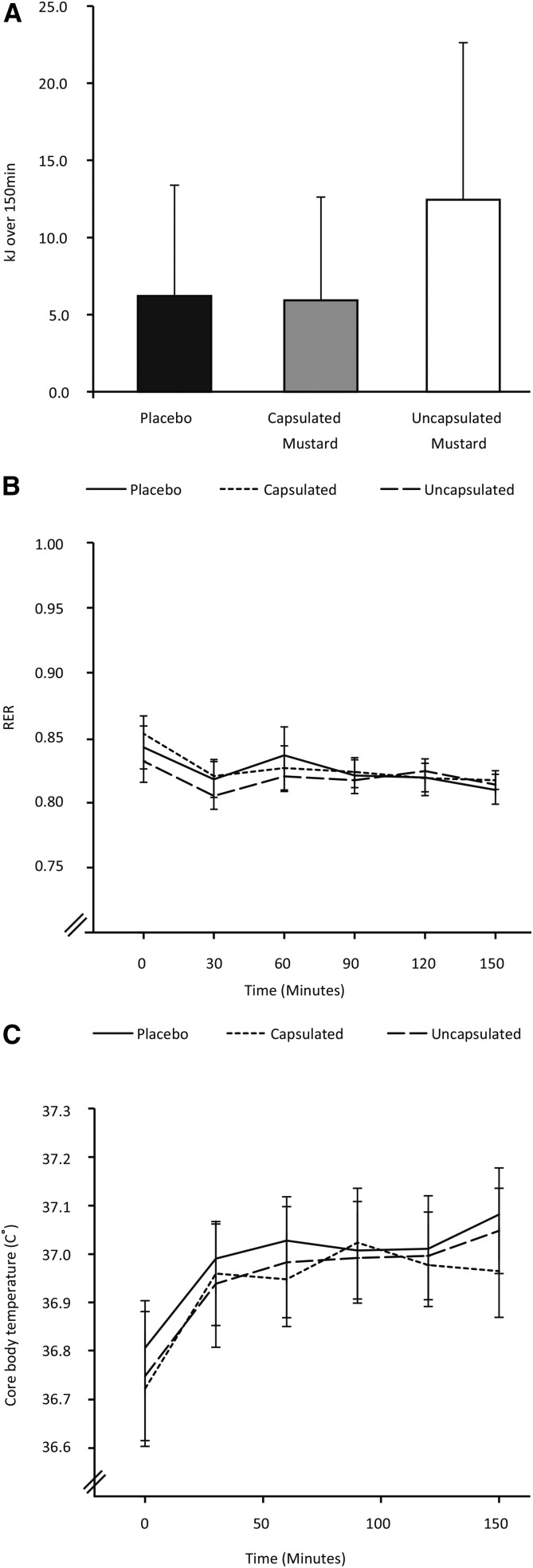
Effect of mustard on energy expenditure and temperature. (A) Cumulative energy expended above basal metabolic rate for 150 min (placebo, *n* = 10; uncapsulated mustard, *n* = 9; capsulated mustard,* n* = 10). Bars represent means and error bars represent SEMs. Capsulated compared with placebo *P* = 0.97, uncapsulated compared with placebo *P* = 0.44, with the use of the Student’s *t* test. (B) Change in RER during 150-min period (placebo, *n* = 10; uncapsulated mustard, *n* = 9; capsulated mustard,* n* = 10). Lines represent mean values and error bars represent SEMs. Repeated-measures ANOVA *P* = 0.04 for the effect of time, *P* = 0.71 for the effect of intervention, and *P* = 0.96 for the interaction time × intervention. (C) Change in core body temperature during the 150-min period (placebo, *n* = 8; uncapsulated mustard, *n* = 7; capsulated mustard, *n* = 7). Lines represent mean values and error bars represent SEMs. Repeated-measures ANOVA *P* < 0.01 for the effect of time, *P* = 0.68 for the effect of intervention, and *P* = 0.92 for the interaction time × intervention. RER, respiratory exchange ratio.

**FIGURE 4:**
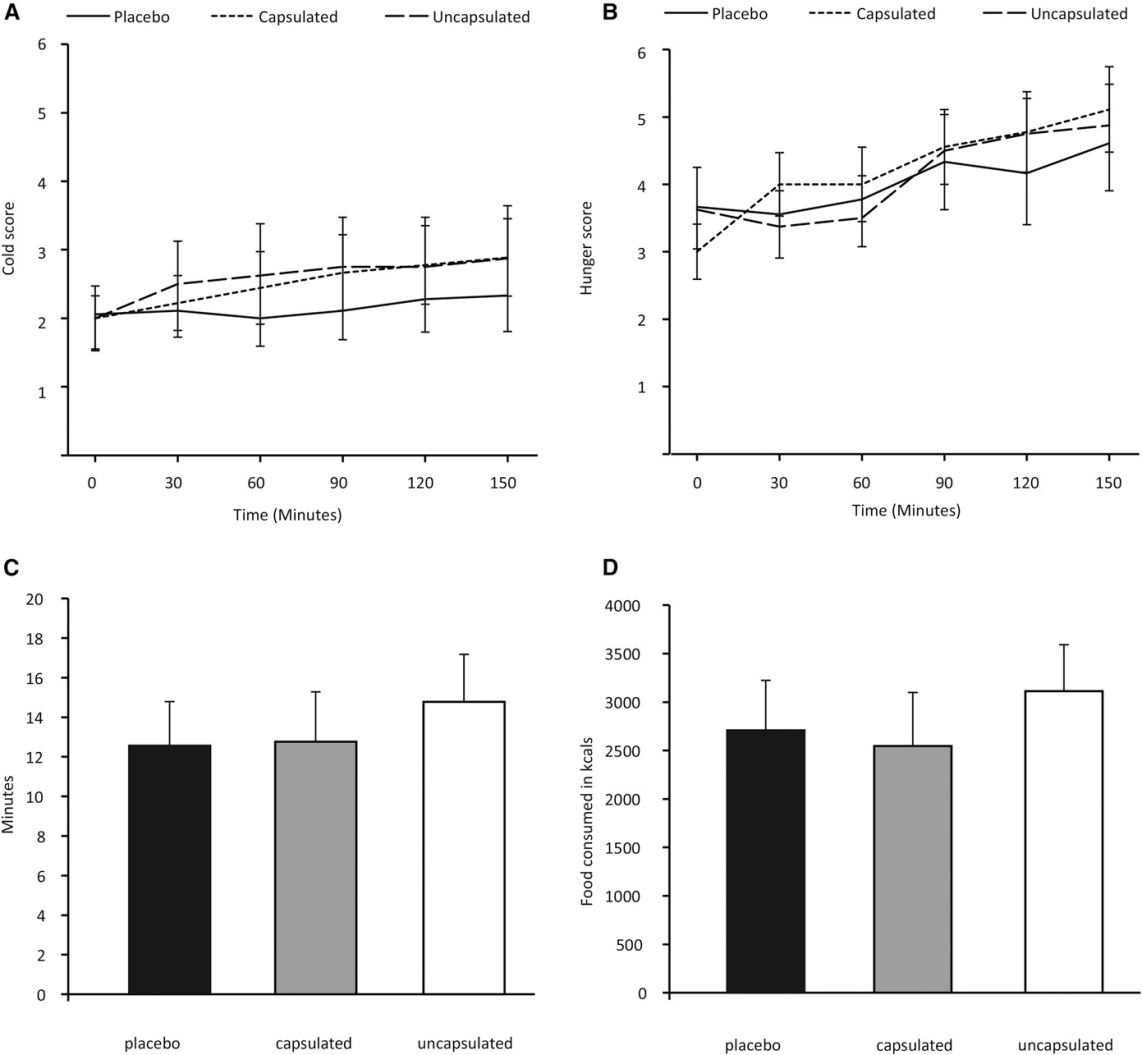
Effect of mustard on cold and hunger scores and food intake. (A) Change in cold scores during the 150-min period (placebo, *n* = 10; uncapsulated mustard, *n* = 9; capsulated mustard,* n* = 10). Lines represent means and error bars represent SEMs. Repeated-measures ANOVA *P* = 0.09 for the effect of time, *P* = 0.65 for the effect of intervention, and *P* = 0.29 for the interaction time × intervention. (B) Change in hunger scores during the 150-min period (placebo, *n* = 10; uncapsulated mustard, *n* = 9; capsulated mustard,* n* = 10). Lines represent means and error bars represent SEMs. Repeated-measures ANOVA *P* < 0.01 for the effect of time, *P* = 0.93 for the effect of intervention, and *P* = 0.32 for the interaction time × intervention. (C) Time spent eating (universal eating monitor) (placebo, *n* = 8; uncapsulated mustard, *n* = 7; capsulated mustard, *n* = 7). Bars represent means and error bars represent SEMs. Capsulated compared with placebo, *P* = 0.83, and uncapsulated compared with placebo, *P* = 0.13 with the use of the Student’s *t* test. (D) Amount of food consumed (universal eating monitor) (placebo, *n* = 10; uncapsulated mustard, *n* = 7; capsulated mustard,* n* = 10). Bars represent means and error bars represent SEMs. Capsulated compared with placebo, *P* = 0.61 and uncapsulated compared with placebo, *P* = 0.37 with the use of the Student’s *t* test.

The measurements of core temperature, ankle temperature, and time spent eating were not performed in all of the subjects because of technical failure of the equipment on several occasions (for these parameters, the number of participants for which the data were available are stated in the legends of **Figures 3** and **4**).

### Indirect calorimetry

After subtracting baseline energy expenditure, energy expended for 150 min postingestion of mustard, either capsulated or uncapsulated, did not differ from the placebo condition (capsulated mustard: 5.9 ± 6.7 kJ, uncapsulated mustard: 12.4 ± 10.2 kJ, placebo: 6.2 ± 7.2 kJ, capsulated mustard compared with placebo *P* = 0.97, uncapsulated mustard compared with placebo *P* = 0.44) (Figure 3A). Mean RER dropped during the 150 min under all 3 conditions (repeated-measures ANOVA *P* = 0.04 for the effect of time), but the ingestion of capsulated or uncapsulated mustard compared with placebo had no relevant effect on RER (Figure 3B).

### Core temperature

Core body temperature during the 150-min period rose significantly under all 3 conditions (repeated-measures ANOVA *P* < 0.01 for the effect of time), but it was not significantly different between the 3 interventions (Figure 3C).

### Cold sensation, hunger, and test meal results

Whole-body cold sensation, rated on a scale from 1 to 10, showed a tendency to increase for 150 min under all 3 conditions (repeated-measures ANOVA *P* = 0.09 for the effect of time). Cold sensation did not differ between the 3 interventions (Figure 4A). Hunger rated on a scale from 1 to 10 increased linearly for the 150 min under all 3 conditions (repeated-measures ANOVA *P* < 0.01 for the effect of time), but it was not affected by the interventions (Figure 4B).

In the test meal, the time spent eating was not significantly different after the ingestion of capsulated or uncapsulated mustard compared with placebo (capsulated mustard: 12.8 ± 2.5 min, uncapsulated mustard: 14.8 ± 2.4 min, placebo: 12.6 ± 2.2 min, capsulated mustard compared with placebo *P* = 0.83, uncapsulated mustard compared with placebo *P* = 0.13) (Figure 4C). Energy consumed in the test meal was not significantly different between the 3 conditions (capsulated mustard: 2547 ± 553 kJ, uncapsulated mustard: 3112 ± 479 kJ, placebo: 2709 ± 515 kJ, capsulated mustard compared with placebo *P* = 0.61, uncapsulated mustard compared with placebo *P* = 0.37) (Figure 4D).

### Biochemistry

**FIGURE 5:**
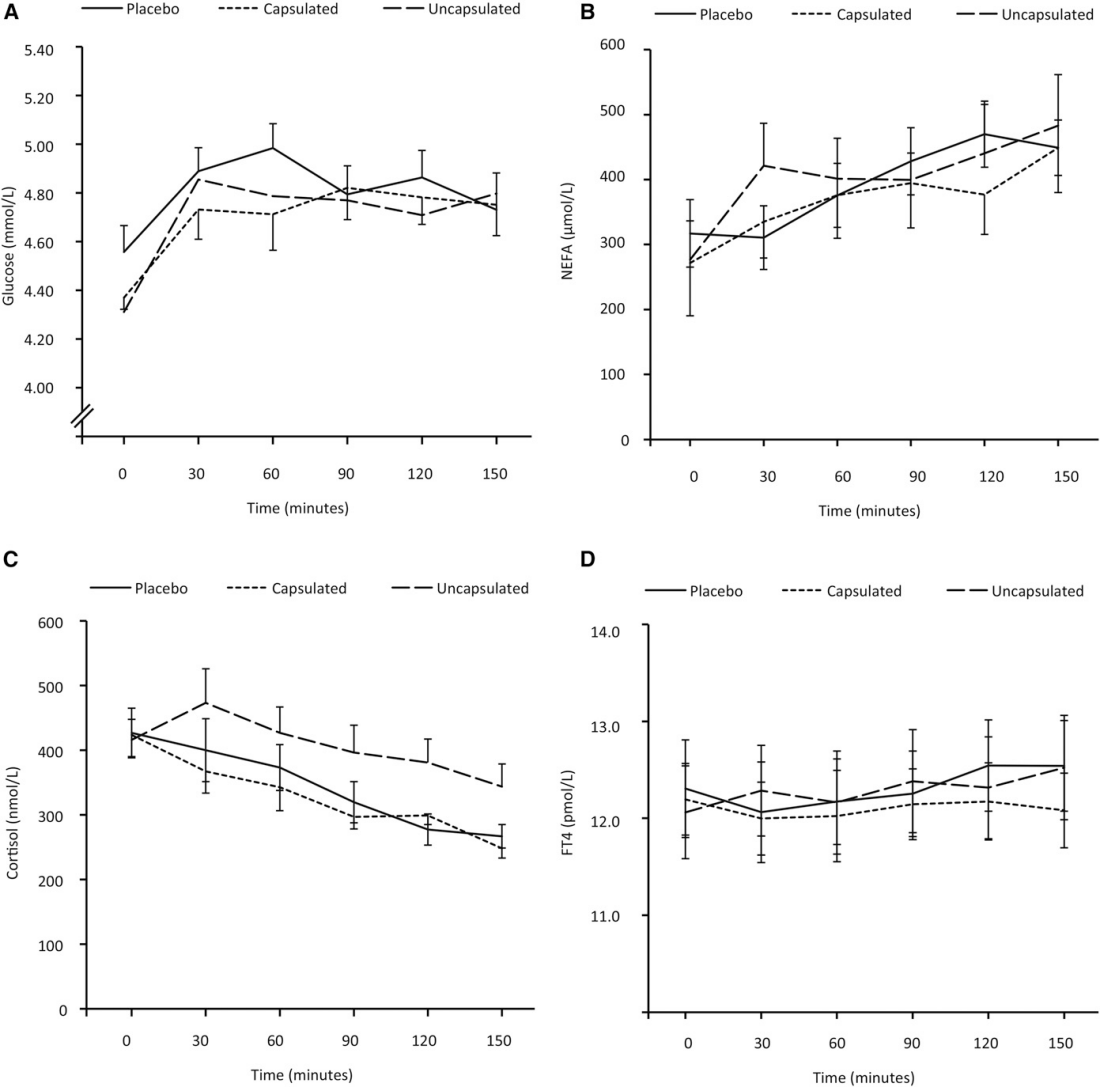
Effect of mustard on plasma metabolic parameters. (A) Change in glucose during the 150-min period. Repeated-measures ANOVA *P* < 0.01 for the effect of time, *P* = 0.29 for the effect of intervention, and *P* = 0.14 for the interaction time × intervention. (B) Change in NEFAs during the 150-min period. Repeated-measures ANOVA *P* < 0.01 for the effect of time, *P* = 0.83 for the effect of intervention, and *P* = 0.15 for the interaction time × intervention. (C) Change in cortisol during the 150-min period. Repeated-measures ANOVA *P* < 0.01 for the effect of time, *P* = 0.15 for the effect of intervention, and *P* = 0.27 for the interaction time × intervention. (D) Change in FT4 during the 150-min period. Repeated-measures ANOVA *P* < 0.01 for the effect of time, *P* = 0.82 for the effect of intervention, and *P* = 0.41 for the interaction time × intervention. Placebo, *n* = 10; uncapsulated mustard, *n* = 9; capsulated mustard, *n* = 10 for all graphs. Lines represent means and error bars represent SEMs. FT4, free thyroxin; NEFA, nonesterified fatty acid.

Plasma glucose concentrations increased during the 150-min period under all 3 conditions (repeated-measures ANOVA *P* < 0.01 for the effect of time), but they were not different between the 3 different conditions (**Figure 5A**). The same was true for the plasma nonesterified fatty acid concentrations (repeated-measures ANOVA *P* < 0.01 for the effect of time) (Figure 5B) and plasma free thyroxin levels (repeated-measures ANOVA *P* < 0.01 for the effect of time) (Figure 5D). Plasma cortisol concentrations dropped linearly during the 150-min period under all 3 conditions (repeated-measures ANOVA *P* < 0.01 for the effect of time), without relevant differences between the 3 conditions (Figure 5C).

## DISCUSSION

In this study, we assessed the thermogenic potential of the pungent component of mustard, AITC. Our proof-of-principle experiment in mice showed that AITC was able to induce a significant increase in energy expenditure. This finding contradicts those of Mori et al. (20), in which no change in energy expenditure was detected after intragastric administration of AITC. The difference between these findings may be explained by the mice in our study being anesthetized and the mice in the Mori et al. study being free moving. Given the modest increase in energy expenditure in response to AITC (10% in our experiment), this can be lost in the variation in energy expenditure in free-moving mice.

We also studied the effect of the ingestion of mustard in humans. We chose to administer the mustard in both capsulated and uncapsulated formulations because we wanted to study whether activation of the TRPA1 receptors in the oral cavity were necessary to induce the hypothesized thermogenic effect. The administration of 10 g of sharp mustard (estimated AITC dose: 16 mg) did not alter energy expenditure during the 150 min postingestion period, however, nor did it change substrate oxidation patterns, cold and hunger sensations, and the amount eaten in the test meal. It also did not significantly change plasma metabolites and hormones. With the experimental setup we used, we were able to detect the subtle changes in these parameters resulting from prolonged fasting, (e.g., the decrease in RER) and those caused by diurnal rhythm (e.g., increase in core body temperature). We cannot rule out that the thermogenic effect of mustard ingestion was so small that we were unable to detect it above the signal of these changes. If that is the case, however, then the induced changes are too small to have a clinically relevant effect on human energy balance.

The current dose of mustard was not capable of inducing a relevant increase in energy expenditure, perhaps because the amount of AITC administered was too low. We used the maximal dose that was tolerable orally, without inducing nausea or other unwanted reactions. As such, providing a higher amount of AITC orally is not feasible.

The absence of an AITC-induced thermogenic response does not negate the possible use of other thermogenic nutrients. Caffeine slightly increases energy expenditure and decreases energy intake, but long-term use causes insensitivity to these effects (6). Green tea, which contains catechin as well as caffeine, may have greater potential to alter energy balance given its ability to alter norepinephrine turnover, but high-quality long-term studies are lacking (7, 8). Capsaicin and its nonpungent counterpart capsiate activate the heat-sensing TRP subfamily V, member 1 (TRPV1); increase energy expenditure; and activate BAT (9).

Specific cold sensing also can be achieved by the TRP agonists cinnamaldehyde, present in cinnamon, and the menthol present in mint plants, which activate TRPA1 and TRPM8, respectively. Like AITC, cinnamaldehyde elicits a cold response with BAT activation and tail vasoconstriction in mice (12). In a pair-fed study, a high-fat, high-sucrose diet caused less weight gain when cinnamaldehyde was mixed into the diet; this may occur through induction of BAT-mediated thermogenesis because the uncoupling protein 1 (UCP1) content of BAT was higher in the treated mice (21). Menthol treatment partially prevented diet-induced obesity and insulin resistance; these effects were not seen in TRPM8 knockout mice (22).

In conclusion, the highest tolerable dose of mustard we were able to use did not elicit a significant thermogenic response in humans. Other potential thermogenic foods may be more suitable for use as weight-controlling agents. These could be studied with the experimental setup described herein because it can accurately detect small but relevant changes in energy expenditure, substrate oxidation, temperature, and food intake.
